# Pediatric Necrobiotic Xanthogranuloma as a Novel Phenotype of IKAROS Gain of Function

**DOI:** 10.1007/s10875-023-01622-4

**Published:** 2023-12-22

**Authors:** Rachel Guess, George Harocopos, Jeffrey J. Bednarski, Lynn M. Hassmann, Tarin M. Bigley

**Affiliations:** 1 Division of Rheumatology/Immunology, Department of Pediatrics, Washington University School of Medicine in St Louis, St. Louis, MO USA; 2 Department of Ophthalmology & Visual Sciences, Washington University School of Medicine, St. Louis, MO USA; 3 Department of Pathology & Immunology, Washington University School of Medicine, St. Louis, MO USA; 4 Division or Hematology/Oncology, Department of Pediatrics, Washington University School of Medicine in St. Louis, St. Louis, MO USA

To the Editor,

The *IKFZ1* (IKAROS) gene has broad roles in T and B lymphocyte development and function. Loss of function variants in the IKAROS gene have been associated with a range of presentations including a spectrum of immunodeficiency, immune dysregulation, and hematologic malignancy [1–3]. Gain of function (GOF) variants in IKAROS were reported in patients presenting with immune dysregulation in the form of allergies, autoimmunity, increased rate of infections, and lymphoproliferation, while somatic variants have been linked to multiple myeloma [4]. We describe a pediatric patient with IKAROS GOF presenting with immune dysregulation and necrobiotic xanthogranuloma (NXG).

A 15-year-old white male presented as a transfer of care with a chronic right orbital mass resulting in right eye vision loss. He first presented at age 5 with fever, rash, and multi-organ failure requiring prolonged hospitalization. He was diagnosed with systemic juvenile idiopathic arthritis (sJIA). Treatment included corticosteroids, hydroxychloroquine, and methotrexate, which induced remission. He was off medications for several years until age 9 when he developed progressive erythematous swelling of the right periocular tissues. Imaging showed a large right inflammatory orbital mass. Biopsy of the right orbital mass demonstrated inflammatory histiocytic infiltration including palisading around necrobiotic areas, negative cultures, negative IgG4 staining, and a CD4:CD8 T cell ratio of 9:1. He was treated with multiple immunosuppressives, including prednisone, hydroxychloroquine, methotrexate, mycophenolate mofetil, adalimumab, abatacept, tocilizumab, intravenous immunoglobulin (IVIG), and rituximab without improvement of the mass. Adalimumab and IVIG were not tolerated due to side effects. He was noted to have moderate seasonal allergies, polyarthralgia, myalgia, and recurrent supra-infections of the right orbital mass. He underwent several surgical procedures on the orbital mass. Repeat biopsies performed 3 years later exhibited xanthogranulomatous inflammation and negative microbial staining. Imaging at age 13 indicated an extensive inflammatory and sclerosing right orbital mass and a lesser involvement of the left orbit and lacrimal gland.

Upon presentation to our center, he was receiving low-dose prednisone and rituximab bimonthly for a chronic orbital mass resulting in facial asymmetry and loss of right-eye vision, presumably due to a combination of compressive optic neuropathy and corneal scarring from proptosis-related exposure (Fig. 1A). His workup demonstrated a negative auto-antibody profile, normal to elevated immunoglobulin levels (while B cell depleted), normal serum IgG4 levels, elevated muscle enzymes, abnormal T cell phenotyping with moderately increased CD4 + memory T cells, and mildly decreased memory B cells (measured after B cell reconstitution) (Supplemental Tables 1–2). The auto-antibody testing may have been affected by rituximab treatment, although was similar prior to treatment apart from an ANA of 1:80. MRI of the right orbital mass revealed diffuse pre- and post-septal enhancing inflammation involving the extraocular muscles, with enhancement and stretching of the optic nerve, and prominent exophthalmos (Fig. 1B). Genetic testing with a primary immunodeficiency panel (Invitae) revealed a pathogenic variant in *IKZF1* on exon 5 at c.548G > A (p.Arg183His), previously documented as a gain of function, as well as a variant of uncertain significance in ITGAM (c.1459C > T (p.Arg487*)) [4]. His sister (age 13) and father were tested, and his sister had the same pathogenic variant in *IKZF1*. On her evaluation, she was found to have seasonal allergies, mildly decreased CD8 + T cells, and increased IgA and IgE (Supplemental Table 3). The family history included multiple females spanning three generations who had arthritis and lung disease resulting in early death, the youngest being his mother at age 34 who had been diagnosed with autoimmune connective tissue disease and right optic neuritis (Fig. 1C).

**Fig. 1:**
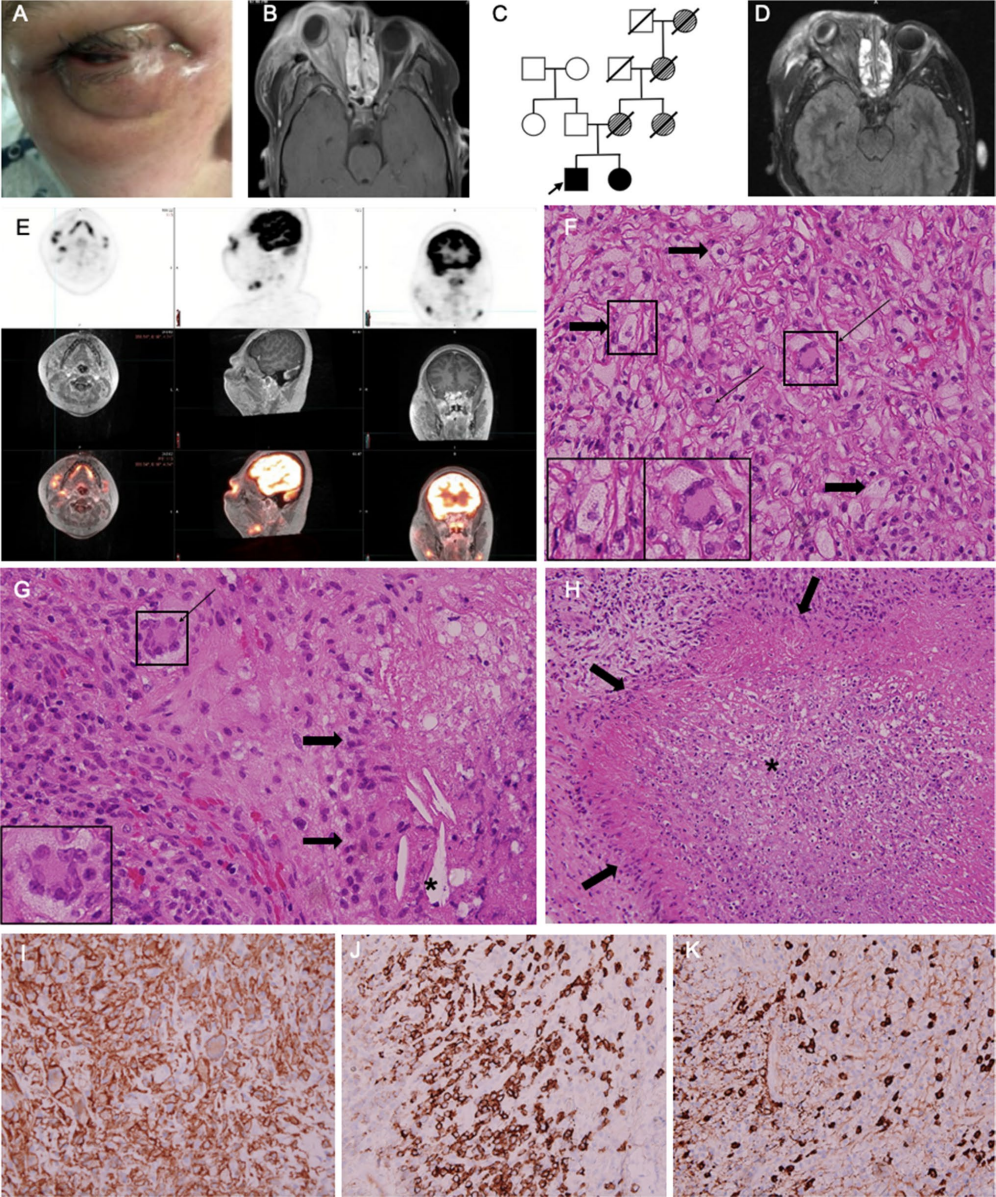
Evaluation of orbital mass and family history of a patient with IKAROS GOF. **A** Image of the patient’s right eye, used with permission from the patient and parent. **B** T2 FLAIR MRI of mass with extensive inflammatory infiltration into the posterior orbital space and proptosis. **C** Partial family pedigree. The arrow represents the proband. Disease with confirmed IKAROS GOF genetic testing is represented in a circle with a black fill while disease not confirmed by genetics is represented in a circle with a striped fill. **D** T2 FLAIR MRI-PET of mass with extension of the inflammatory mass following a COVID infection. **E** MRI-PET with PET-avid lymph nodes in the right pre-auricular area, right upper soft palate, and right anterior cervical chain. **F** “Foamy” histiocytes (thick arrows) and multiple Touton giant cells (thin arrows) (H&E stain, original magnification 400 ×). **G** Classic Touton giant cell (thin arrow) with palisading macrophages (thick arrows) and cholesterol clefts (star) (H&E stain, original magnification 400 ×). **H** Palisading histiocytes (thick arrows) around a necrobiotic area (star), (H&E stain, original magnification 200 ×). **I** CD163 immunostain highlighting abundant histiocytes, including Touton giant cells (original magnification 400 ×). **J** CD138 immunostain highlighting plasma cell–rich areas (original magnification 400 ×). **K** IgG4 immunostain highlighting up to 100 plasma cells per high-power field, comprising about 25% of the total plasma cell population, indicating increased expression (original magnification 400 ×)

Our patient was initiated on sirolimus; however, he had progression of the bilateral orbital masses following a COVID infection. MRI also demonstrated enhancing, infiltrative tissue advancing into the right parotid gland, right cervical lymph nodes, and right soft palate (Fig. 1D). Due to concern for malignant transformation, a PET scan was obtained (Fig. 1E). This revealed a hypermetabolic infiltrative mass with a maximum standardized uptake value (SUV) of 13, favoring an inflammatory process over malignancy. Biopsies of the right orbital mass, right parotid gland, and lymph node were consistent with NXG, characterized by granulomatous inflammation with “foamy” histiocytes and Touton giant cells (Fig. 1F), and palisading histiocytes surrounding areas of necrobiosis (Fig. 1 G and H). There was also a mixed lymphoplasmacytic inflammation including some lymphoid follicles, along with scattered eosinophils. No vasculitis was seen. The abundant histiocytes were highlighted by CD163 immunostain (Fig. 1I). The lesion was plasma cell–rich as evidenced by CD138 immunostaining (Fig. 1J) with increased IgG4 expression (Fig. 1K). Immunostaining for CD1a and BRAF V600E mutation were negative (Supplemental Fig. 1A and 1B), as was microbial staining and culture. The histo-morphology did not demonstrate any evidence of lymphoma, nor did any of the additional immunohistochemical markers that were obtained for lymphoma work-up, while kappa and lambda in situ hybridization confirmed a polytypic mixture of plasma cells, in a 2:1 ratio. Bone marrow biopsy showed hypocellular trilineage hematopoiesis with no blasts. Lymph node flow cytometry was negative for malignancy. Serum protein electrophoresis was normal.

NXG is classified as a non-Langerhans cell histiocytosis. NXG is an inflammatory mass typically spurred by an underlying immune dysregulation such as malignancy or autoimmunity [5–7]. It often presents as a slowly progressive anterior orbital mass bilaterally [5]. As described above for our patient, NXG histology is characterized by necrobiotic zones with palisading histiocytes, Touton giant cells, cholesterol clefts, and lymphoplasmacytic infiltrate [5]. It most commonly occurs in older individuals, with average age of diagnosis being 61. NXG is often seen in association with paraproteinemia or multiple myeloma [5]. NXG is typically responsive to immunosuppression and treatment of underlying disease. In a systemic review of 175 patients, the most common therapies were IVIG, corticosteroids, alkylating agents, and corticosteroid combination therapies [6]. We therefore started our patient on a combination therapy of cyclophosphamide (500 mg oral once weekly × 4 weeks), dexamethasone (20 mg twice daily on days 1 to 4 and 12 to 15), and high-dose IVIG (1 g/kg monthly). After two cycles of therapy, he had clinical improvement of the lesion and a small reduction in size based on MRI (Supplemental Fig. 1C&D). He will proceed to hematopoietic stem cell transplant after a reduction in the size of his NXG.

In summary, we describe a pediatric case of IKAROS GOF resulting in immune dysregulation with multiple phenotypic expressions including allergies, susceptibility to bacterial infections, autoimmunity diagnosed as sJIA in early childhood, myositis, and, most notably, NXG resulting in facial asymmetry and vision loss. Although family members with the disease were deceased before we were able to perform genetic testing, we can presume based on history, that he is the fourth generation with autoimmunity related to IKAROS GOF, with increasingly severe disease in each generation.

To our knowledge, at age 9 our patient is the youngest to present with NXG and the first to be diagnosed with NXG in relation to an underlying inborn error of immunity (IEI) [7]. Juvenile xanthogranuloma, a related non-Langerhans cell histiocytosis, has been associated with germline *NF1*, *NF2*, and *WASP* variants and somatic *BRAF*, *MAP2K1*, *MET*, *KRAS*, *ALK*, *PI3CKD*, and *CSF1R* variants, some of which are genes also associated with IEIs [8, 9]. IKAROS GOF leads to broad immune dysregulation, including early terminal differentiation of T lymphocytes and decreased memory B cells. There appears to be a tendency towards lymphoproliferation in IKAROS GOF patients, with lymphadenopathy and multiple myeloma previously described. Our patient had progressive orbital NXG without evidence of malignancy, suggesting it occurred secondary to his underlying immune dysregulation. This further exemplifies the hematologic abnormalities observed in IKAROS GOF. His lesion was IgG4 enriched and histiocyte-predominant. A previously described IKAROS GOF patient had histologic features of the histiocyte disorder, Rosai-Dorfman Disease [4]. Together, these findings suggest a role of IKAROS in histiocyte homeostasis. In conclusion, this case demonstrates a phenotypic expansion of IKAROS GOF to include systemic life-threatening hyperinflammation along with subsequent development of NXG at a very young age and highlights the treatment-resistant nature of this disease.

### Supplementary Information

Below is the link to the electronic supplementary material.

Supplementary file1 (DOCX 424 KB)

## Data Availability

The authors confirm that the data supporting the findings of this study are available within the article and/or its supplementary materials.
