# Supplementary material for: Pediatric Necrobiotic Xanthogranuloma as a Novel Phenotype of IKAROS Gain of Function
Source: J Clin Immunol. 2023 Dec 22;44(1):19. doi: 10.1007/s10875-023-01622-4 (PMC10739487; doi:10.1007/s10875-023-01622-4)

**Supplemental Figure 1. Additional histologic and radiologic characterization of NXG in a patient with IKAROS GOF. (A)** Negative CD1a immunostain (original magnification 200x). **(B)** Negative immunostain for the BRAF V600E mutation (original magnification 200x). **(C)** Patient image after 2 cycles of NXG treatment. (D) T2 FLAIR MRI post-2 cycles of treatment with cyclosphosphamide, dexamethasone, and IVIG with small decrease in mass size and proptosis.

**Supplemental Table 1**. Relevant antibody testing, liver/muscle enzyme levels, and immunoglobulin classes at presentation.

**Supplemental Table 2**. Lymphocyte subsets after B cell reconstitution. T- and B-cell phenotyping, and T-cell spectratyping.

**Supplemental Table 3**. Relevant labs for patient’s 13-year-old sister who carries the IKAROS GOF variant.


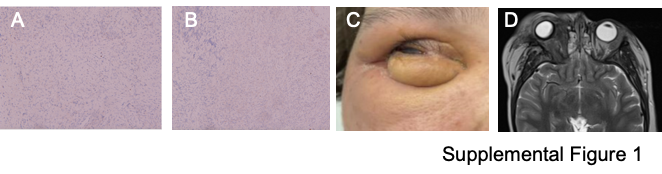


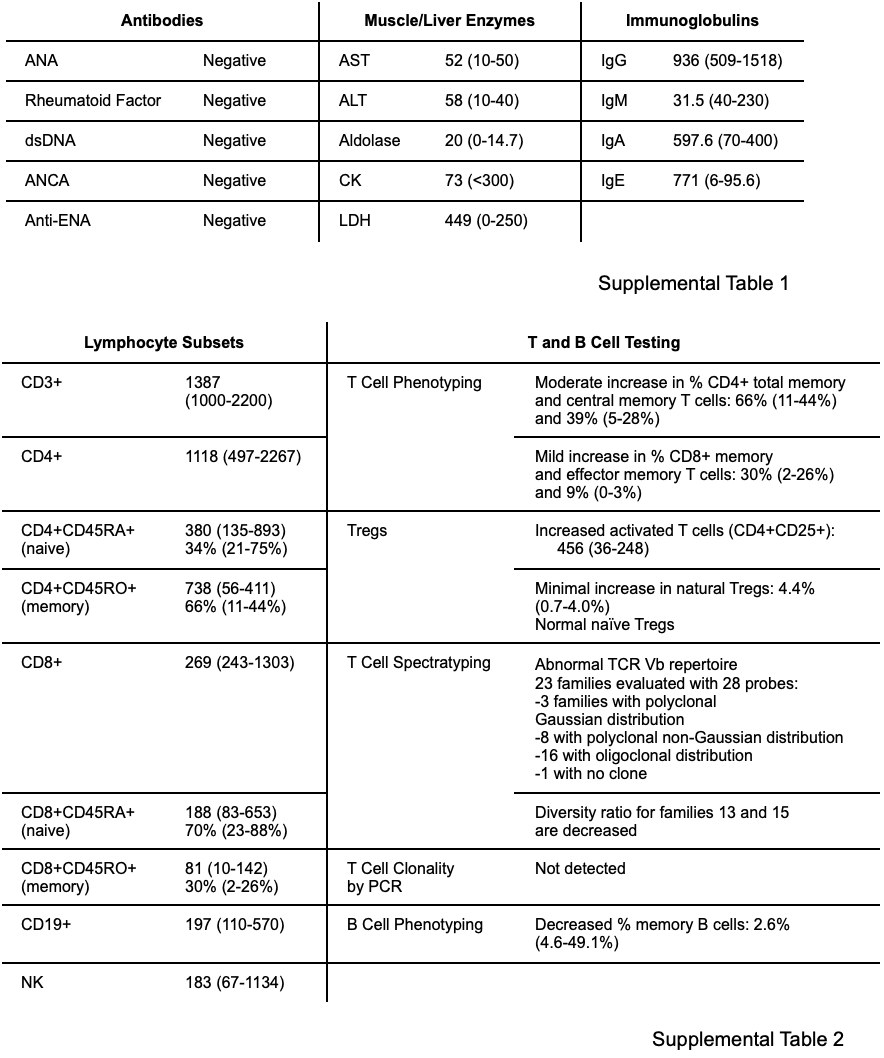


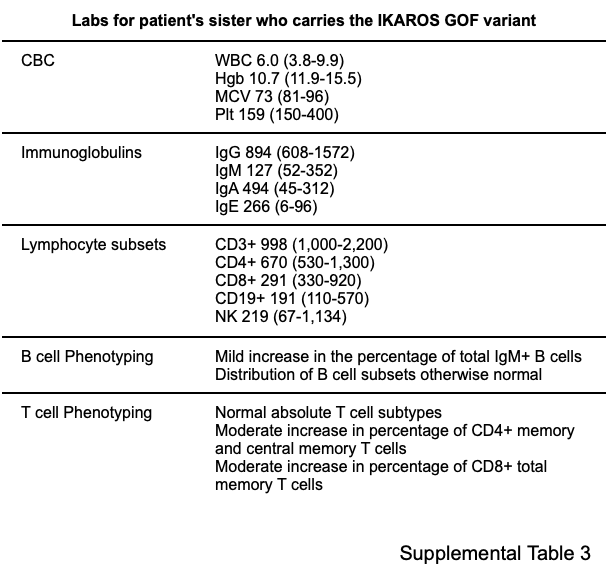

Supplement: Supplementary file 1 — Supplementary file1 (DOCX 424 KB) [file 10875_2023_1622_MOESM1_ESM.docx]
